# Optimizing Laser Powder Bed Fusion Parameters for IN-738LC by Response Surface Method

**DOI:** 10.3390/ma13214879

**Published:** 2020-10-30

**Authors:** Mireia Vilanova, Rubén Escribano-García, Teresa Guraya, Maria San Sebastian

**Affiliations:** 1 LORTEK Technological Centre, Basque Research and Technology Alliance (BRTA), Arranomendia Kalea 4A, 20240 Ordizia, Spain; rescribano@lortek.es (R.E.-G.); msansebastian@lortek.es (M.S.S.); 2 Department of Mining and Metallurgical Engineering and Materials Science, University of the Basque Country UPV/EHU, Rafael Moreno “Pitxitxi”, 2, 48013 Bilbao, Spain; teresa.guraya@ehu.eus

**Keywords:** laser powder bed fusion (LPBF), Inconel 738LC, response surface method (RSM), process parameter optimization, cracking

## Abstract

A method to find the optimum process parameters for manufacturing nickel-based superalloy Inconel 738LC by laser powder bed fusion (LPBF) technology is presented. This material is known to form cracks during its processing by LPBF technology; thus, process parameters have to be optimized to get a high quality product. In this work, the objective of the optimization was to obtain samples with fewer pores and cracks. A design of experiments (DoE) technique was implemented to define the reduced set of samples. Each sample was manufactured by LPBF with a specific combination of laser power, laser scan speed, hatch distance and scan strategy parameters. Using the porosity and crack density results obtained from the DoE samples, quadratic models were fitted, which allowed identifying the optimal working point by applying the response surface method (RSM). Finally, five samples with the predicted optimal processing parameters were fabricated. The examination of these samples showed that it was possible to manufacture IN738LC samples free of cracks and with a porosity percentage below 0.1%. Therefore, it was demonstrated that RSM is suitable for obtaining optimum process parameters for IN738LC alloy manufacturing by LPBF technology.

## 1. Introduction

Laser powder bed fusion (LPBF) is an additive manufacturing technology in which a laser melts successive powder layers in order to build the final part [1]. Some of the LPBF process parameters are laser power (*P*), hatch distance (*h*), laser scan speed (*v*), layer thickness (*t*), baseplate preheating temperature and laser scan strategy (ϴ). The latter refers to the rotation of successive layers during the manufacturing process. Combining some of the process parameters, a key factor for LPBF technology known as energy density can be calculated, as shown in Equation (1). This factor indicates the energy provided to the material during manufacturing process [2]:

(1) $$E_{d}=\frac{P}{v\cdot h\cdot t}$$

Compared with conventional manufacturing processes (cast and wrought), LPBF technology offers some advantages, such as design freedom, reduced weight of parts, processing of complex parts, manufacturing of near-net-shape components and reduction of waste material [3]. Despite these advantages, the presence of defects such as pores and cracks in the manufactured final parts is a drawback for the implementation of this technology in the industry [4]. In particular, the existence of porosity is attributed to different mechanisms: insufficient energy density, porosity in raw material, excessive energy density or large spatter particles [5]. Pores formed by an insufficient energy density usually have irregular morphology due to the insufficient melting of the powder particles; nevertheless, porosity formed by the other mechanisms usually presents as spherical. For instance, powder particles’ inner porosity may be entrapped in the manufactured final parts due to the rapid solidification during LPBF process. Additionally, when the applied energy density is too high, the melt pool becomes unstable, inducing the formation of spherical pores at the bottom of the melt pool which are known as keyhole defects [2].

There is a wide range of materials processable by LPBF technology, including aluminum (Al) alloys [6,7,8], titanium (Ti) alloys [9], stainless steels [10,11], cobalt (Co) alloys [12], cupper (Cu) alloys [13] and nickel (Ni) alloys [14,15]. Among the nickel (Ni)-based superalloys, Inconel 738LC (IN738LC) is of huge interest for components of the hottest section of land-based and aeronautic gas turbine engines because of its corrosion resistance and creep properties at high temperatures [16]. The microstructure of the superalloy consists of a face centered cubic (FCC) matrix strengthened by the precipitation of the ordered second phase γ’ which has a nominal composition of Ni_3_(Al, Ti) [17]. The corrosion resistance of the alloy is achieved by the formation of the Cr_2_O_3_ protective layer at the surface, whereas the outstanding mechanical properties at high temperatures are obtained by the precipitation of the γ’ phase.

Nevertheless, IN738LC alloy is not considered processable by LPBF technology because of its high susceptibility to cracking during manufacturing process. In the literature, the cracking susceptibility of IN738LC is explained by three main mechanisms: liquation cracking, solidification cracking and strain age cracking [18,19]. Liquation cracking occurs when the material is heated just below the liquidus temperature where some low melting point components—carbides, γ/γ’ eutectic, borides, etc.—could suffer partial or total melting. In the case of solidification cracking, it occurs at the final stages of solidification when the liquid fraction present in the material is around 6–10% [20]. This liquid is enriched in some elements, such as zirconium (Zr) and boron (B), which decrease the solidification temperature of the alloy known as the solidus. Actually, the remaining liquid fraction accumulates in the intergranular zones, causing stress during its solidification, which acts as an initial point of cracking. While liquation and solidification cracking are liquid state mechanisms, strain age cracking is solid state mechanism which occurs during the precipitation of the γ’ phase. As the mismatch between Ni matrix and γ’ precipitates is below 1%, precipitation of the second phase occurs extremely rapidly, involving large amount of stress which could cause the separation of grain boundaries. This type of cracking tends to take place in Ni alloys with Al and Ti amounts higher than 4.5% (wt%) [21].

Some authors have focused on eliminating the cracking phenomenon in Ni superalloys using different approaches. Rickenbacher et al. [17] reduced crack density in the IN738LC superalloy through the optimization of LPBF process parameters. However, in order to obtain manufactured parts without cracks, they conducted HIP (hot isostatic pressing) as a post-processing step. Xu et al. [18] asserted that using a sufficiently high preheating temperature, it is possible to decrease the alloy’s thermal range and to change the microstructure from columnar to equiaxial grains. This microstructural change implies a more homogeneous distribution of the liquid along grains, reducing or even eliminating the formation of cracks. In fact, they manufactured crack-free IN738LC parts by applying a preheating temperature of 1050 °C. Cloots et al. [19] suggested that the formation of cracks in the IN738LC superalloy occurs by solidification cracking mechanism due to the presence of a thin liquid film along the investigated cracks. They confirmed by atom probe tomography technique that the liquid film along the cracks was rich in Zr and B elements. They concluded that in order to eliminate cracking, it would be necessary to minimize as much as possible the content of B and remove Zr from the alloy totally. Finally, Carter et al. [22] suggested that by controlling scan strategy of manufactured samples, it was possible to significantly reduce cracking density. Actually, they studied island and simple scan strategies and determined that there were differences in the crack density values of the samples manufactured with each one of the strategies.

Due to the formation of defects in IN738LC parts, it is challenging trying to optimize process parameters. Furthermore, taking into account the manufacturing process variables and all their possible combinations, it would be unaffordable to find the optimum process parameters by means of trial and error method. For this reason, Perevoshchikova et al. [23] used Doehlert’s design to optimize laser power, laser scan speed and hatch distance process parameters to manufacture samples with minimum porosity.

In this work, the authors propose the application of the response surface method (RSM) to optimize laser power, hatch distance, scan speed and scan strategy process parameters with a reduced number of trials. RSM consists of a design of experiments (DoE), polynomial model fitting and optimization with a combination of desirabilities and the steepest ascent method. The response surface method has been tested in several fields over the decades; however, the method does not ensure the expected results, so experimental verification is needed. The potential problems of RSM are related to the modeling error that could be induced by the measurement challenges. In the case of additive manufacturing, another problem could be caused by the sample’s position on the platform. In addition, one of the limitations of RSM may be the number of inputs, because when inputs are increased the number of samples for building the models is also exponentially increased. It may be important to point out that without previous information about the material, it will be necessary to build more than one model to set the appropriate process parameter range.

In the field of additive manufacturing, several researchers have used RSM for process parameter optimization. Wang et al. [24] investigated the influences of some LPBF process parameters on the microstructure and mechanical properties of manufactured samples by RSM. They concluded that it was possible to increase the mechanical properties by the optimization of process parameters through applying RSM. In addition, Deng et al. [25] manufactured 316L stainless steel samples with high density and low roughness using RSM for the process optimization. Terner et al. [26] optimized the laser power and scanning speed processing parameters by using RSM to manufacture Co-CrMo samples with a residual porosity and an increase in the hardness of the material. In this study, the optimization was carried out to obtain samples with minimum porosity and no cracks. The method followed minimizes manufacturing time, material waste, post processing tasks and evaluation time.

## 2. Methodology

### 2.1. Response Surface Method

RSM is a method that uses DoE, regression models and desirability functions [27]. The objective of RSM is to explore the relations between input variables and response variables, and to find the optimum working point using the minimum number of trials. DoE is a collection of techniques (full factorial, central composite, box-Behnken, etc.) to define a reduced set of trials whose results depend on input factors. Currently, these methodologies are widely used to perform multi-objective optimizations of manufacturing processes [28,29]. DoE determines the number of cases, combinations, randomization, replication and blocking of the factors to study cause–effect relationships with a certain degree of confidence.

The number of combinations with four factors is 3^4^ = 81 considering three levels per factor. This kind of design is called full three-level design (also called 3k) because it considers all possible combinations of factors with three levels. In this case, manufacturing 81 cases (or cubes) is very expensive and time consuming in terms of manufacturing and measuring. The number of samples can be reduced to 15 using a central composite design (CCD), which is an adequate fractional factorial design to fit quadratic models.

After sample manufacturing, outputs are measured for all the cases. Then, a polynomial model is adjusted for each output using the inputs and the results of the 15 samples from the CCD:

(2) $$Y=f\left(x_{1},x_{2},\;\ldots ,\;x_{n}\right)$$

where *Y* is a calculated output, *f* is a polynomial function and *x_i_* are the inputs of the regression model. The quadratic regression model is a polynomial function that is widely used because it considers non-linear effects and allows finding combined influences of inputs taken in pairs. The general form of the quadratic models is as follows:

(3) $$Y=b_{0}+\overset{n}{\underset{i=1}{\sum }}b_{i}\cdot x_{i}+\overset{n}{\underset{i=1}{\sum }}b_{ii}\cdot x_{i}^{2}+\overset{n-1}{\underset{i=1}{\sum }}\overset{n}{\underset{j=i+1}{\sum }}b_{ij}\cdot x_{i}\cdot x_{j}+e$$

where *b*_0_ is the independent term; the first and second summations are linear and quadratic terms; the third summation is the cross product of all input factors; and *e* is the error. Analysis of variance (ANOVA) can be used to evaluate the adjustment of the regression models to the experimental data.

In multi-objective optimization, responses can conflict with each other, which means that a solution can provide the optimal response for some objectives and poor responses for the rest. In these cases, generally no unique result achieves the best solution to all objectives at the same time. Instead, several Pareto-efficient solutions cannot be improved in any objective without worsening other ones. Harrington proposed evaluating the overall response by applying the following expression [27,30]:

(4) $$D={\left(d_{1}\cdot d_{2}\cdot \ldots \cdot d_{m}\right)}^{1/m}$$

where *D* is the overall desirability for a certain solution (inputs combination), *m* is the number of outputs and *d_i_* the desirability for the output *i*. The desirability of each output is defined depending on objective type (maximum, minimum, target, etc.). In this study, the objectives are to minimize the porosity (ϕ) and crack density (CD). These objectives desirability is defined as follows:

(5) $$d=\{\begin{array}{cc} 1 & if\;Y\leq L_{min}\;\\ {\left(\frac{L_{max}-Y}{L_{max}-L_{min}}\right)}^{s} & if\;L_{min}<Y\leq L_{max}\\ 0 & if\;Y\geq L_{max} \end{array}$$

where *L_min_* and *L_max_* are the limit values and *s* is an exponent specified by the user. The exponent s defines the shape of the desirability function and is equal to one in this study because both outputs are considered equally important.

### 2.2. Materials

Considering the specifications of LPBF technology, gas atomized IN738LC powder was supplied by Aubert and Duval as feedstock material. Powder chemical composition, detailed in Table 1, was measured by induced coupled plasma (ICP) technique and by LECO for carbon and oxygen elements.

**Figure 1:**
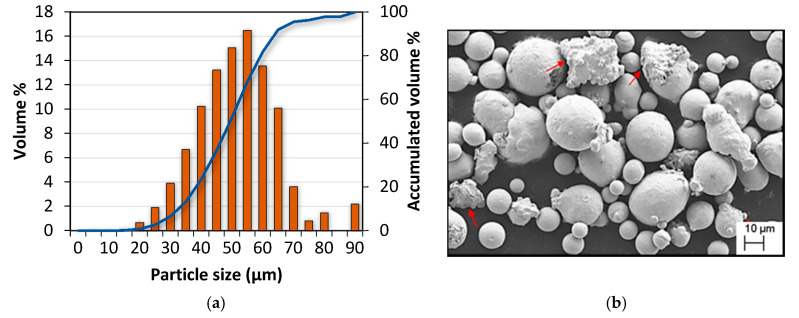
(**a**) Particle size distribution of IN738LC; (**b**) SEM image of the powder particles showing differences in size and presence of satellites and irregular particles (red arrows).

Powder particle size distribution (PSD) measured by image analysis is shown in Figure 1a, whereas D10 and D90 values were 32.57 and 64.06 µm respectively. Powder particles’ morphology was evaluated by scanning electron microscopy (SEM, Zeiss, Jena, Germany), and as presented in Figure 1b the morphology was generally spherical, although some irregular particles and satellites may be seen, which are indicated by red arrows.

**Figure 2:**
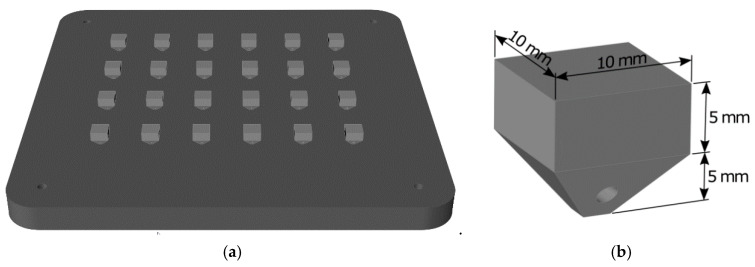
(**a**) Twenty-four manufactured cubes; (**b**) dimensions of the samples.

Samples were manufactured by LPBF in a RenAM 500Q Renishaw machine which employs four Yb-fiber lasers in a continuous way with a maximum power of 500 W and a laser spot size of 85 µm. For the design of experiments, 24 samples were manufactured in the same baseplate (Figure 2a) with dimensions described in Figure 2b. All the samples were cut in building direction (x–z plane) to investigate the cracking phenomenon.

In order to define process parameters’ ranges for IN738LC, the parameters used by Renishaw for IN718 material (which is a Ni based alloy processable by LPBF) and the ones optimized by other authors for IN738LC [23] were taken into account. In this case, four input factors were optimized: laser power, laser scan speed, hatch distance and scan strategy. Table 2 summarizes the factors, symbols, ranges and units used in this work. Other process parameters were kept constant, such as layer thickness (60 µm) and preheating temperature (170 °C).

Samples were prepared metallographically by grinding up to 2500 µm SiC paper and polishing with 6, 3 and 1 µm diamond paste. Lastly, the manufactured samples were characterized by light microscope (GX51 Olympus) and a Zeiss Ultra Plus FEG-SEM (Zeiss, Jena, Germany).

### 2.3. Porosity and Cracking Quantification

Porosity and crack density were measured to evaluate the numbers of defects in all manufactured samples. On the one hand, porosity quantification was performed by image analysis using five images of each sample captured with the light microscope. A threshold value was applied in order to separate defects from consolidated material [26,31]. This was done by trying different threshold values and finally selecting the one with which the noise of the image was eliminated without eliminating the smallest pores of 10 µm. With respect to crack quantification, crack densities were measured following the methodology described by Carter et al. [32]. Finally, crack density value was calculated dividing the total crack length by total area.

## 3. Results and Discussion

### 3.1. DoE and Sample Characterization

Table 3 summarizes the input parameters applied between the specified ranges according to central composite design (CCD) and the measured outputs. As previously explained, 15 samples were enough to build the model; however, any number of samples greater than 15 allows the entire polynomial to be fitted and that is why 24 samples were manufactured. All cubes were manufactured in one baseplate and were randomized to avoid bias due to non-controllable factors, such as argon flow inside LPBF chamber. The randomization also avoids bias during measurement and calculation outputs. In addition, Table 3 presents the output results and defect type found in each sample: LOF—lack-of-fusion defects; P—pores; and C—cracks.

**Figure 3:**
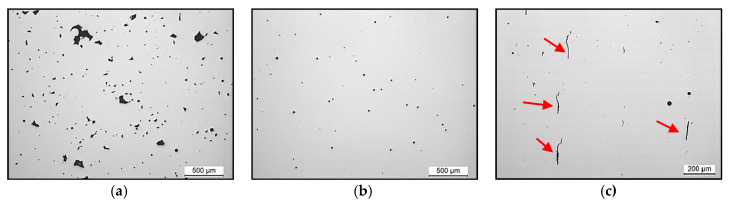
IN738LC samples showing different type of defects: (**a**) lack of fusion, (**b**) pores and (**c**) cracks (indicated by an arrow) and pores.

Figure 3 shows different types of defects observed in IN738LC samples manufactured based on the DoE. The defects were classified mainly as: (a) lack of fusion due to low energy density, (b) pores due to excessive energy density and (c) cracks because of the set of parameters selected.

### 3.2. Influence of Process Parameters on Defectology

After analyzing the manufactured samples, an optimal energy density range between 38 and 80 J/mm^3^ was described to avoid lack of fusion defects. However, the parameters selected for sample number two induced an energy density higher than 100 J/mm^3^, which produced an out of plane distortion in the sample. As this effect damaged the wiper during the manufacturing process, sample number two was dismissed.

**Figure 4:**
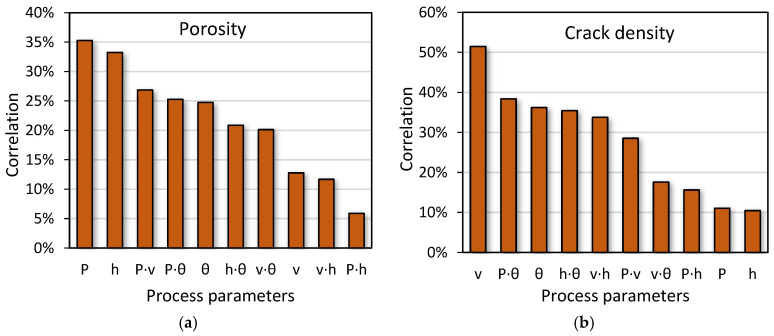
Influences of process parameters on defect formation. (**a**) Porosity; (**b**) cracking.

After sample characterization and defect quantification, it was possible to determine the correlation of each process parameter and each combination (cross products) with the formation of pores and cracks, as shown in Figure 4. Correlation is determined by the degree of influence of one variable on another, and it is expressed in absolute value, which means that the correlation between process parameters and defectology may be positive or negative. In the case of porosity formation, laser power and hatch distance are the most influential process parameters because they are related to the appearance of a lack of fusion defects. In fact, when laser power is too low, powder particles are not completely melted and a lack of fusion defects is observed [33]. In the same way, if hatch distance is too high, a proper overlap is not ensured, which induces a lack of fusion defects in the melted material [34]. Therefore, the most effective way to reduce sample porosity would be by changing the laser power or hatch distance values.

When the formation of cracks is considered, it is shown that the most influential parameters are laser scan speed and the combination between laser power and scan strategy. Firstly, Cloots et al. [19] stated that variation of scan speed affects melt pool morphology, which influences the critical temperature range (CTR) of the material. In fact, they assure that the volume of material that is in the CTR has a significant importance on crack formation. Thus, these authors concluded that lower scan speeds increased melt pool depth and the volume of material in the CTR, which favored crack development. Secondly, Carter et al. [22] revealed the connection between scan strategies and formation of cracks. These authors showed that depending on the scan strategy used, it was possible to change samples grain structure, which is related to grain misorientation. Actually, they observed by electron backscattered diffraction (EBSD) maps that the majority of cracks appear in grain boundary regions with high misorientation level. Therefore, the most effective way to reduce sample crack density would be by changing the laser scan speed value or scan strategy selected.

Models to predict optimum process parameters were built using inputs and outputs from Table 3 by means of “R” statistical package [35]. Two kinds of quadratic models were built: the first one using all the manufactured samples and the second one only using samples without lack of fusion defects, which means that samples 5, 7 and 15 presented in Table 3 were dismissed. Finally, the second option was selected because the prediction error was lower than in the first case. This fact could be due to a massive appearance of pores in the form of lack-of-fusion defects. The porosity related to lack-of-fusion presented a maximum value of 13.74%, while the maximum value for porosity without lack-of-fusion defects was 0.40%, which is significantly lower. This may indicate that the excessive variation between porosity values of samples with and without lack-of-fusion defects could increase the model’s error, and because of that, it was decided to dismiss the samples with lack-of-fusion defects. Furthermore, the quadratic models were fitted (Equations (6) and (7)) using normalized data in order to study the importance of process parameters and their combinations. In that way, terms with higher coefficients in absolute value have more influence on the output than terms with lower coefficients.

(6) $$\begin{array}{ll} \varphi = & -0.5088-0.7363\cdot P\cdot v+0.492\cdot v^{2}-1.1766\cdot h\\ & +0.9205\cdot v\cdot h+0.9615\cdot h^{2}+1.0268\cdot \theta -1.0944\cdot \theta^{2} \end{array}$$

(7) $$\begin{array}{ll} \mathrm{CD}= & 0.0112-0.7517\cdot P+0.3391\cdot P\cdot v+0.39371\cdot v^{2}-0.63914\cdot h\\ & +0.73062\cdot P\cdot h+0.49322\cdot v\cdot h+0.884\cdot h^{2}+0.46287\cdot P\cdot \theta \\ & -1.26285\cdot v\cdot \theta -0.64007\cdot h\cdot \theta +0.91777\cdot \theta^{2} \end{array}$$

Additionally, mean absolute error (MAE) and root mean square error (RMSE) were calculated according to Equations (8) and (9).

(8) $$MAE=\frac{1}{k}\cdot \overset{k}{\underset{i=1}{\sum }}\left|e_{i}\right|=\frac{1}{k}\cdot \overset{k}{\underset{i=1}{\sum }}\left|Y_{i\;exp}-Y_{i\;model}\right|$$

(9) $$RMSE={\left(\frac{1}{k}\cdot \overset{k}{\underset{i=1}{\sum }}e_{i}^{2}\right)}^{0.5}={\left(\frac{1}{k}\cdot \overset{k}{\underset{i=1}{\sum }}{\left(Y_{i\;exp}-Y_{i\;model}\right)}^{2}\right)}^{0.5}$$

Table 4 provides p-values and MAE and RMSE errors; p-values are low in both cases, which means the quadratic models were statistically significant. The crack density model presents a better p-value and lower errors than the porosity model. This revels porosity has much more variability, which could be provoked by the manufacturing process.

Once the models were obtained and evaluated, it was possible to find the optimum working point. Package “desirability” [36], available in “R,” was used to calculate the porosity, crack density and overall desirabilities (see Equation (5)). The determination of the working point with maximum desirability was achieved by application of the steepest ascent method [37]. The objectives of the optimization were to minimize crack density and porosity. The results of the optimization are listed in Table 5.

The optimal working point (Table 5) was used to prepare a new manufacturing run. Five samples were fabricated with the same optimal parameters to validate the method. Table 6 compares the calculated values with the experimental ones. It was necessary to round some input parameters to set up the LPBF machine.

As expected, the calculated porosity had a certain error compared with the experimental one, although all the samples presented residual porosity below 0.1%. Those results were obtained because the selected combination of process parameters induced sufficient energy density to prevent lack-of-fusion defects, but avoided keyhole pores formed by excessive energy density. Additionally, no cracks were found in the manufactured samples, which indicates that the process parameters selected by the model reduce the crack sensitivity of IN-738LC superalloy.

**Figure 5:**
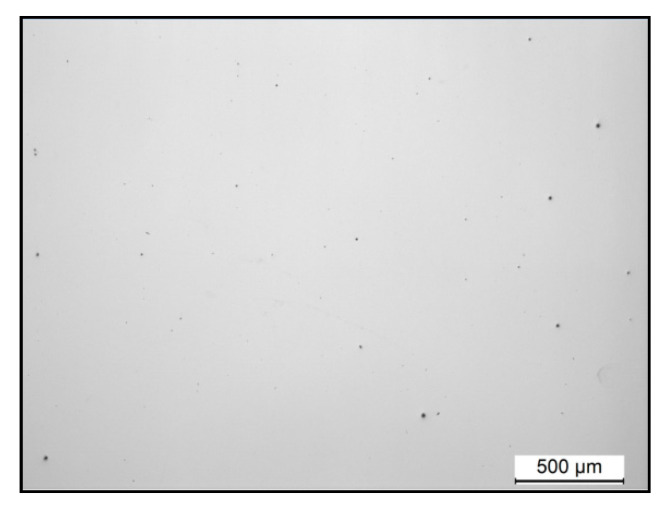
IN738LC sample manufactured with predicted optimum process parameters.

As shown in Figure 5, the sample manufactured with optimal process parameters had reduced porosity and no cracks. Consequently, it is demonstrated that using the parameters obtained from Equations (6) and (7), it is possible to manufacture samples of IN738LC superalloy without significant defects.

## 4. Conclusions

This study investigated the use of models to obtain components fabricated by LPBF technology free of pores and cracks. Firstly, a widely used method, known as the response surface method, was successfully applied with the objective of reducing the number of experiments and selecting the process parameters for IN738LC alloy. In particular, the optimized LPBF process parameters were laser scan speed, laser power, hatch distance and scan strategy.

Secondly, it was possible to determine the influence of each process parameter on the formation of pores and cracks by correlation analysis. It was concluded that laser power and hatch space were the most influential factors in terms of pore formation. However, with respect to crack development, scan speed is the process parameter with the highest impact due to the fact that it may alter the material’s volume in the critical temperature range.

Finally, a design of experiments with 24 samples was defined. However, the results of just 20 samples were used to build the models, because samples with lack-of-fusion defect were dismissed. The process parameters determined by the model were used for the manufacturing of IN738LC superalloy by LPBF technology. Using the selected parameters, five samples were manufactured to experimentally validate the proposed method. After analyzing these samples, we verified the possibility of manufacturing samples with reduced porosity and no cracks.

Therefore, in the present work, it was demonstrated that despite the high cracking susceptibility of IN738LC superalloy, it is possible to manufacture samples through LPBF technology without cracks using the suitable process parameters obtained by RSM.

## Figures and Tables

**Table 1:** Composition of the IN738LC superalloy used for manufacturing.

**Element**	**Cr**	**Co**	**W**	**Ti**	**Al**	**Ta**	**Mo**	**Fe**	**Nb**
wt%	15.8	8.6	2.7	3.3	3.6	1.8	1.8	0.04	0.8

**Element**	**Si**	**Zr**	**Mn**	**B**	**C**	**O**	**N**	**Ni**	
wt%	0.02	0.04	0.002	0.0009	0.1	0.02	0.006	Bal.	

**Table 2:** Factors, ranges and units of the design of experiments.

Factor	Symbol	Range		Unit
		Min	Max	
Laser power	P	180	280	W
Laser scan speed	v	700	1100	mm/s
Hatch distance	h	0.08	0.12	mm
Scan strategy	θ	0	90	°

**Table 3:** Design of experiments and results.

n	Inputs				Outputs		Defect
	P	v	h	θ	ϕ	CD *	Type
**1**	180	700	0.08	0	0.15	0.00	P
**2**	280	700	0.08	0	-	-	
**3**	180	1100	0.08	0	0.23	0.00	P
**4**	280	1100	0.08	0	0.09	0.00	P
**5**	180	700	0.12	0	1.26	0.16	LOF/P/C
**6**	280	700	0.12	0	0.11	0.16	P/C
**7**	180	1100	0.12	0	13.74	0.00	LOF
**8**	280	1100	0.12	0	0.17	0.00	P
**9**	180	700	0.08	90	0.15	0.64	P/C
**10**	280	700	0.08	90	0.13	0.45	P/C
**11**	180	1100	0.08	90	0.21	0.04	P/C
**12**	280	1100	0.08	90	0.10	0.08	P/C
**13**	180	700	0.12	90	0.24	0.10	P/C
**14**	280	700	0.12	90	0.10	0.68	P/C
**15**	180	1100	0.12	90	12.21	0.33	LOF
**16**	280	1100	0.12	90	0.40	0.11	P/C
**17**	180	900	0.10	67	0.27	0.03	P/C
**18**	280	900	0.10	67	0.04	0.00	P
**19**	230	700	0.10	67	0.09	0.06	P/C
**20**	230	1100	0.10	67	0.25	0.01	P/C
**21**	230	900	0.08	67	0.16	0.00	P
**22**	230	900	0.12	67	0.20	0.26	P/C
**23**	230	900	0.10	0	0.11	0.00	P
**24**	230	900	0.10	90	0.07	0.09	P/C

* Crack density measurement specified in this work doesn’t detect cracks smaller than 10 µm.

**Table 4:** *p*-value and errors of the quadratic models.

Defect Type	*p*-Value	MAE	RMSE
ϕ	0.07	10.1%	13.6%
CD	0.02	3.6%	5.5%

**Table 5:** Optimization results.

Process Parameter and Defect Type	Target	Value	Desirability
**P**	In range	272.60 W	1.00
**v**	In range	799.50 mm/s	1.00
**h**	In range	0.11 mm	1.00
**θ**	In range	1.54°	1.00
**ϕ**	Minimize	0.07%	0.94
**CD**	Minimize	0.00 mm/mm^2^	1.00
Overall desirability:			0.97

**Table 6:** Optimization results comparison.

Process Parameter and Defect Type	Calculated	Experimental	Difference
**P (W)**	272.6	273.0	0.4
**v (mm/s)**	799.5	800.0	0.5
**h (mm)**	0.11	0.11	0.00
**θ (°)**	1.54	0.00	−1.54
**ϕ (%)**	0.07	0.09 ± 0.01	0.02
**CD (mm^−1^)**	0.00	0.00	0.00
